# Reaction-Diffusion-Delay Model for EPO/TNF-*α *Interaction in articular cartilage lesion abatement

**DOI:** 10.1186/1745-6150-7-9

**Published:** 2012-02-21

**Authors:** Jason M Graham, Bruce P Ayati, Lei Ding, Prem S Ramakrishnan, James A Martin

**Affiliations:** 1 Department of Mathematics/Program in Applied Mathematical and Computational Sciences, University of Iowa, Iowa City, Iowa, USA; 2 Department of Orthopaedics and Rehabilitation, University of Iowa Hospitals and Clinics, Iowa City, Iowa, USA

## Abstract

**Background:**

Injuries to articular cartilage result in the development of lesions that form on the surface of the cartilage. Such lesions are associated with articular cartilage degeneration and osteoarthritis. The typical injury response often causes collateral damage, primarily an effect of inflammation, which results in the spread of lesions beyond the region where the initial injury occurs.

**Results and discussion:**

We present a minimal mathematical model based on known mechanisms to investigate the spread and abatement of such lesions. The first case corresponds to the parameter values listed in Table 1, while the second case has parameter values as in Table 2. In particular we represent the "balancing act" between pro-inflammatory and anti-inflammatory cytokines that is hypothesized to be a principal mechanism in the expansion properties of cartilage damage during the typical injury response. We present preliminary results of *in vitro *studies that confirm the anti-inflammatory activities of the cytokine erythropoietin (EPO). We assume that the diffusion of cytokines determine the spatial behavior of injury response and lesion expansion so that a reaction diffusion system involving chemical species and chondrocyte cell state population densities is a natural way to represent cartilage injury response. We present computational results using the mathematical model showing that our representation is successful in capturing much of the interesting spatial behavior of injury associated lesion development and abatement in articular cartilage. Further, we discuss the use of this model to study the possibility of using EPO as a therapy for reducing the amount of inflammation induced collateral damage to cartilage during the typical injury response.

**Conclusions:**

The mathematical model presented herein suggests that not only are anti-inflammatory cy-tokines, such as EPO necessary to prevent chondrocytes signaled by pro-inflammatory cytokines from entering apoptosis, they may also influence how chondrocytes respond to signaling by pro-inflammatory cytokines.

**Reviewers:**

This paper has been reviewed by Yang Kuang, James Faeder and Anna Marciniak-Czochra.

**Table 1:** Model Parameter Values for Results in Figure 5

Table of Parameter Values Corresponding to Simulations in Figure 5			
**Parameter**	**Value**	**Units**	**Reason**

*D*_*R*_	0.1	$\frac{\text{c}{\text{m}}^{2}}{\text{day}}$	Determined from [13]

*D*_*M*_	0.05	$\frac{\text{c}{\text{m}}^{2}}{\text{day}}$	Determined from [13]

*D*_*F*_	0.05	$\frac{\text{c}{\text{m}}^{2}}{\text{day}}$	Determined from [13]

*D*_*P*_	0.005	$\frac{\text{c}{\text{m}}^{2}}{\text{day}}$	Determined from [13]

*δ*_*R*_	0.01	$\frac{1}{\text{day}}$	Approximated

*δ*_*M*_	0.6	$\frac{1}{\text{day}}$	Approximated

*δ*_***F***_	0.6	$\frac{1}{\text{day}}$	Approximated

*δ*_***P***_	0.0087	$\frac{1}{\text{day}}$	Approximated

*δ*_***U***_	0.0001	$\frac{1}{\text{day}}$	Approximated

*σ*_*R*_	0.0001	$\frac{\text{micromolar}\cdot \text{c}{\text{m}}^{2}}{\text{day}\cdot \text{cells}}$	Approximated

*σ*_*M*_	0.00001	$\frac{\text{micromolar}\cdot \text{c}{\text{m}}^{2}}{\text{day}\cdot \text{cells}}$	Approximated

*σ*_*F*_	0.0001	$\frac{\text{micromolar}\cdot \text{c}{\text{m}}^{2}}{\text{day}\cdot \text{cells}}$	Approximated

*σ*_*P*_	0	$\frac{\text{micromolar}\cdot \text{c}{\text{m}}^{2}}{\text{day}\cdot \text{cells}}$	Case with no anti-inflammatory response

Λ	10	micromolar	Approximated

*λ*_*R*_	10	micromolar	Approximated

*λ*_*M*_	10	micromolar	Approximated

*λ*_*F*_	10	micromolar	Approximated

*λ*_*P*_	10	micromolar	Approximated

*α*	0	$\frac{1}{\text{day}}$	Case with no anti-inflammatory response

*β*_1_	100	$\frac{1}{\text{day}}$	Approximated

*Β*_2_	50	$\frac{1}{\text{day}}$	Approximated

*γ*	10	$\frac{1}{\text{day}}$	Approximated

*ν*	0.5	$\frac{1}{\text{day}}$	Approximated

$\mu_{S_{A}}$	1	$\frac{1}{\text{day}}$	Approximated

$\mu_{D_{N}}$	0.5	$\frac{1}{\text{day}}$	Approximated

*τ*_1_	0.5	days	Taken from [5]

*τ*_2_	1	days	Taken from [5]

**Table 2:** Model Parameter Values for Results in Figure 6

Table of Parameter Values Corresponding to Simulations in Figure 6			
**Parameter**	**Value**	**Units**	**Reason**

*D*_*R*_	0.1	$\frac{\text{c}{\text{m}}^{2}}{\text{day}}$	Determined from [13]

*D*_*M*_	0.05	$\frac{\text{c}{\text{m}}^{2}}{\text{day}}$	Determined from [13]

*D*_*F*_	0.05	$\frac{\text{c}{\text{m}}^{2}}{\text{day}}$	Determined from [13]

*DP*	0.005	$\frac{\text{c}{\text{m}}^{2}}{\text{day}}$	Determined from [13]

*δ*_*R*_	0.01	$\frac{1}{\text{day}}$	Approximated

*δ*_*M*_	0.6	$\frac{1}{\text{day}}$	Approximated

*δ*_*F*_	0.6	$\frac{1}{\text{day}}$	Approximated

*δ*_*P*_	0.0087	$\frac{1}{\text{day}}$	Approximated

*δ*_*U*_	0.0001	$\frac{1}{\text{day}}$	Approximated

*σ*_*R*_	0.0001	$\frac{\text{micromolar}\cdot \text{c}{\text{m}}^{2}}{\text{day}\cdot \text{cells}}$	Approximated

*σ*_*M*_	0.00001	$\frac{\text{micromolar}\cdot \text{c}{\text{m}}^{2}}{\text{day}\cdot \text{cells}}$	Approximated

*σ*_*F*_	0.0001	$\frac{\text{micromolar}\cdot \text{c}{\text{m}}^{2}}{\text{day}\cdot \text{cells}}$	Approximated

*σ*_*P*_	0.001	$\frac{\text{micromolar}\cdot \text{c}{\text{m}}^{2}}{\text{day}\cdot \text{cells}}$	Approximated

Λ	10	micromolar	Approximated

*λ*_*R*_	10	micromolar	Approximated

*λ*_*M*_	10	micromolar	Approximated

*λ*_*F*_	10	micromolar	Approximated

*λ*_*P*_	10	micromolar	Approximated

α	10	$\frac{1}{\text{day}}$	Approximated

*β*_1_	100	$\frac{1}{\text{day}}$	Approximated

*β*_2_	50	$\frac{1}{\text{day}}$	Approximated

*γ*	10	$\frac{1}{\text{day}}$	Approximated

*ν*	0.5	$\frac{1}{\text{day}}$	Approximated

$\mu_{S_{A}}$	1	$\frac{1}{\text{day}}$	Approximated

$\mu_{D_{N}}$	0.5	$\frac{1}{\text{day}}$	Approximated

*τ*_1_	0.5	days	Taken from [5]

*τ*_2_	1	days	Taken from [5]

## Background

Articular cartilage is composed of cells known as chondrocytes. Mechanical stress and injury is known to kill (via necrosis) chondrocytes and results in the formation of lesions on the cartilage surface [1-4]. The limited capacity of chondrocytes to self-repair, together with certain aspects of the typical injury response such as inflammation, can cause the spread of lesions and development of osteoarthritis. Recent research [5] suggests that inflammatory cytokines such as tumor necrosis factor *α *(TNF-*α*) play a significant role in causing the spread of cartilage lesions. Anti-inflammatory cytokines such as erythropoietin (EPO) play an antagonistic role to TNF-*α*, limiting the area over which a lesion can spread by counteracting some of the effects of inflammation [5]. Moreover, the authors in [5] describe the potential use of EPO as a therapy for cartilage injury and lesion abatement

**Figure 1:**
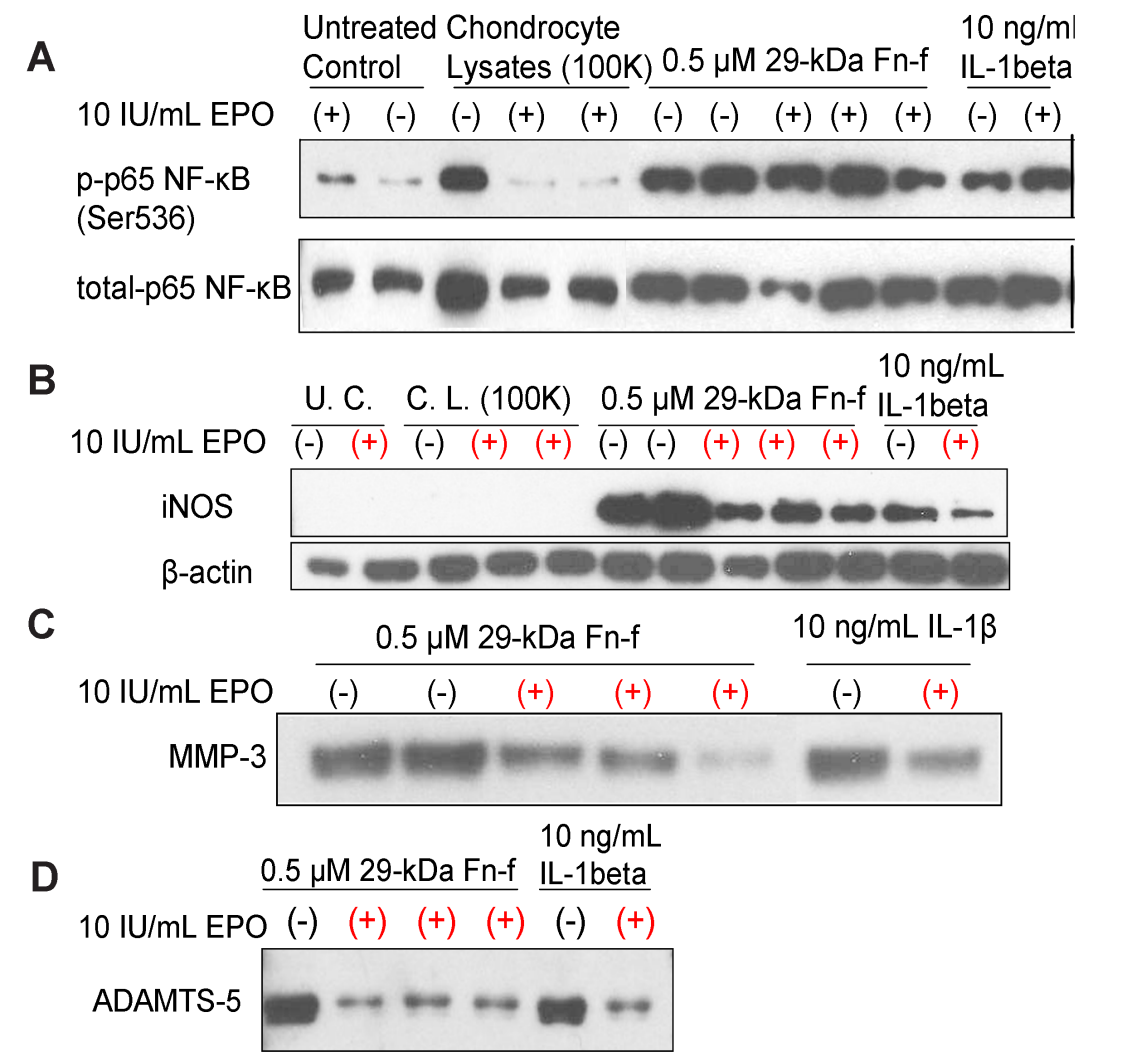
**Effects of EPO on chondrocyte responses to alarmins and IL-1 beta**. Immunoblot analyses are shown for phospho- and total nuclear factor kappa-B (NF-kB), a pro-inflammatory transcription factor (A), inducible nitric oxide synthase (iNOS), a catabolic signaling factor (B), matrix metalloproteinase-3 (MMP-3) (C), and a disintegrin and metalloproteinase domain with thrombospondin repeats (ADAMTS-5) (D), which cause cartilage matrix degeneration. Primary cultures of bovine chondro-cytes (1 × 10^6 ^cells/well) were treated with 10 IU/ml EPO for 24 hours, then challenged for an additional 24 hours with the indicated concentrations of purified 29 kDa fibronectin fragment, a known extracellular matrix DAMP, or IL-1*β*, a pro-inflammatory cytokine, or cell lysates containing multiple cellular DAMPS. EPO treatment suppressed catabolic responses induced by all three treatments.

The full clinical implementation of an EPO derived therapy for osteoarthritis may be a difficult task. One initial step is to establish experimentally that EPO suppresses inflammation *in vitro*. The potential for EPO to suppress catabolic and inflammatory responses of chondrocytes to various alarmins, i.e. molecules that trigger the innate immune response, was explored in a cell culture model (Figure 1). Bovine articular chondrocytes in serum-free culture were pre-treated with EPO and then exposed to alarmins in the form of cell lysates or the 29 kDa fibronectin fragment (Fn-f). Interleukin-1*β *(IL-1*β*) was used as a control. Cell lysates were made by repeated freeze-thawing of excess cells from the same culture. Culture media were collected for immunoblot analysis of matrix metalloproteinase-3 (MMP-3) and A disintegrin and metalloproteinase with thrombospondin motifs 5 (ADAMTS-5). Cell layers were extracted for analysis of nuclear factor *κ*B (NF-*κ*B) and inducible nitric oxide synthase (iNOS). The results revealed that lysates, Fn-f, and IL-1*β *induced NF-*κ*B phosphorylation (Figure 1A). Fn-f, and IL-1*β *but not lysates also induced iNOS, MMP-3 and ADAMTS-5 expression (Figure 1B, C, D). In the case of NF- B, EPO treatment blocked activation by lysates, but not by Fn-f or IL-1*β*. EPO also dramatically reduced iNOS, MMP-3, and ADAMTS-5 expression in response to Fn-f and IL-1*β*. These results underscore the ability of EPO to counter injury effects on multiple pathways.

**Figure 2:**
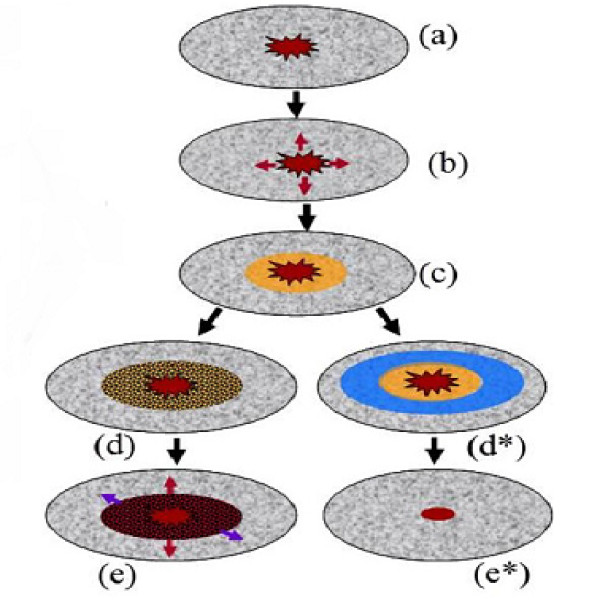
**Injury response**. When a wound occurs DAMPs promote production of TNF-*α *(b), an initially viable penumbra (yellow in (c)) of cells becomes EPOR active. These cells are in danger of death which can result in the spread of injury as seen on the left in (d) and (e). However, EPO can counteract the effects of TNF-*α *and prevent spread of the lesion (d∗) and (e∗).

Figure 2 illustrates the balance between pro-inflammatory (e.g. TNF-*α*) and anti-inflammatory (e.g. EPO) cytokines during the typical injury response. Damage due to chemical or mechanical stress initiates production of alarmins, such as damage associated molecular pattern molecules (DAMPs) [5-7]. These alarmins trigger production of pro-inflammatory cytokines such as TNF-*α *by cells near the initial injury site (see (b) in figure 2) [5]. Thus there is a penumbra ((c) in figure 2) of "sick" cells formed at the boundary of the initial injury as a result of inflammation. While still viable, these cells are at risk of dying themselves due to the presence of the pro-inflammatory cytokines, resulting in the spread of injury as shown in (d)-(e) in figure 2. However, cells far enough from the injury are able to produce anti-inflammatory cytokines such as EPO to check the spread of injury and promote healing. This is shown (d∗)-(e∗) in figure 2. It is also suggested in [5] that TNF-*α *acts in such a way to limit EPO signaling. Thus there is a critical balance between pro-inflammatory and anti-inflammatory cytokines which determines the spreading properties of an articular cartilage lesion associated with injury. Moreover, the authors in [5] suggest that introduction of exogeneous EPO at an early time after an initial injury could be an effective therapy for minimizing the amount of collateral damage to cartilage that occurs as a result of inflammation.

The goal of this work is to study via mathematical modeling the EPO/TNF-*α *interaction described above, and in particular to provide a conceptual framework for a truly mechanistic understanding of some aspects of cartilage injury closely related to lesion expansion. Herein we develop a model capable of simulating the scenario illustrated in figure 2. Moreover, the model is developed as a potential for studying the use EPO or other anti-inflammatory cytokines as a therapy for lesion abatement. The type of model, and in particular the one described below, in this article is of value as it provides a link between observed phenomena and the mechanisms involved in driving that phenomena. Models such as those presented below can also help to generate hypotheses and suggest experiments or fundamental quantities to be measured. More practically, mathematical modeling such as we use in this paper could provide quick and inexpensive initial screening of potential therapies.

## Methods

In this section we describe the mathematical model developed to represent the biological interactions of chondrocytes and cytokines described in [5]. We aim for a minimal model based on known mechanisms considered to be the dominant factors in articular cartilage lesion abatement. By a minimal model we mean one in which the removal of any component results in behavior that is inconsistent with the typical injury responses in cartilage as discussed in [5]. We note that the model described below can be expanded or modified to include further interactions as new results from experiment and observation dictate. In particular, further chemical pathways such as interleukin-1 *β *(IL1-*β*) can easily be incorporated *if necessary*. The chemical species included below are chosen for their functionality that is their action on chondrocytes during injury response. Hence they can be replaced with any other chemical species whose effects on chondrocytes are functionally the same. The chondrocyte cell states described below represent the biological actions of the cells in response to signaling by a particular cytokine during the typical injury response. These actions are assumed to be analogous to those of other cell types, for which aspects of innate immune response, such as local inflammation considered in this paper, are well established.

**Figure 3:**
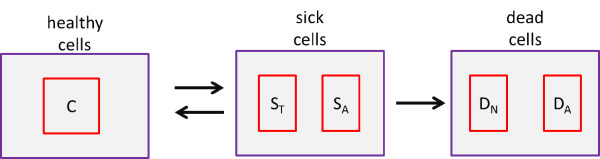
**States of chondrocytes during stereotypical injury response**. Healthy cells (*C*) become sick. Typically a healthy cell will first become catabolic (*S*_*T*_) and either enter apoptosis (*D*_*A*_), or become EPOR active (*S*_*A*_). EPOR active cells may be saved by EPO to become healthy once again, or die (apoptosis).

We consider a population of chondrocytes fixed in matrix, which is typical of articular cartilage. Sub-populations of chondrocytes are assumed to exist in different states corresponding to the chemical signals being received by the cells during injury response. Figure 3 shows the states in which subpopulations of chondrocytes may exist. We refer to the normal state of a subpopulation of chondrocytes as the healthy state. We denote by *C *the population density (cells per unit area) of healthy chondrocytes at a given time and location. As a result of inflammation and injury healthy chondrocytes can enter into a "sick" state. Cells in this state are at risk death (via apoptosis) unless their signaling by TNF-*α *is somehow limited. We consider two subpopulations of cells in the sick state. We denote by *S*_*T *_the population density of cells in the "catabolic" state. Catabolic cells are chondrocytes that have been signaled by alarmins and are capable of synthesizing TNF-*α *and other cytokines associated with inflammation. Healthy cells signaled by DAMPs or TNF-*α *enter into the catabolic state and begin to synthesize TNF-*α *and produce reactive oxygen species (ROS). Catabolic cells that are signaled by TNF-*α *express a receptor (EPOR) for EPO and make up the subpopulation of sick cells we refer to as EPOR active. It should be noted that there is a time delay of 8-12 hours before a cell expresses the EPO receptor after being signaled to become EPOR active [5]. We denote the population density of EPOR active cells by *S*_*A*_. Since EPOR active cells express a receptor for EPO, they may switch back to the healthy state if signaled by EPO. However, as discussed in [5] TNF-*α *limits production of EPO. Thus there is a balance between EPO and TNF-*α *that determines the spreading behavior of cartilage lesions. The catabolic and EPOR active cells together make up the population of cells forming the penumbra as illustrated in figure 2. We also consider a "dead" state for subpopulations of chondrocytes. This includes necrotic cells *D*_*N *_and apoptotic cells *D*_*A*_. We note that for the purposes considered herein that apoptotic cells do not feed back into the system and thus are not explicitly represented in the mathematical model. Due to the abrupt nature of the injury, we assume that the initial injury results in necrosis of cells at the injury site. Furthermore, we assume that cell death due to secondary cytokine-induced injury is strictly through apoptosis. The reasoning here is that necrosis is a nonspecific event that occurs in cases of severe pathological cell and tissue damage, whereas secondary cytokine-induced injury corresponds with a physiologic form of cell death used to remove cells in a more orderly and regulated fashion and there is evidence that often, this is via apoptosis [8]. While these may be simplifying assumption, it is not entirely clear what type of cell death dominates in osteoarthritis, and there is some evidence to support our assumptions [8]. Necrotic cells release alarmins such as DAMPs that initiate the injury response. Either catabolic or EPOR active cells become apoptotic if signaled by inflammatory cytokines.

**Figure 4:**
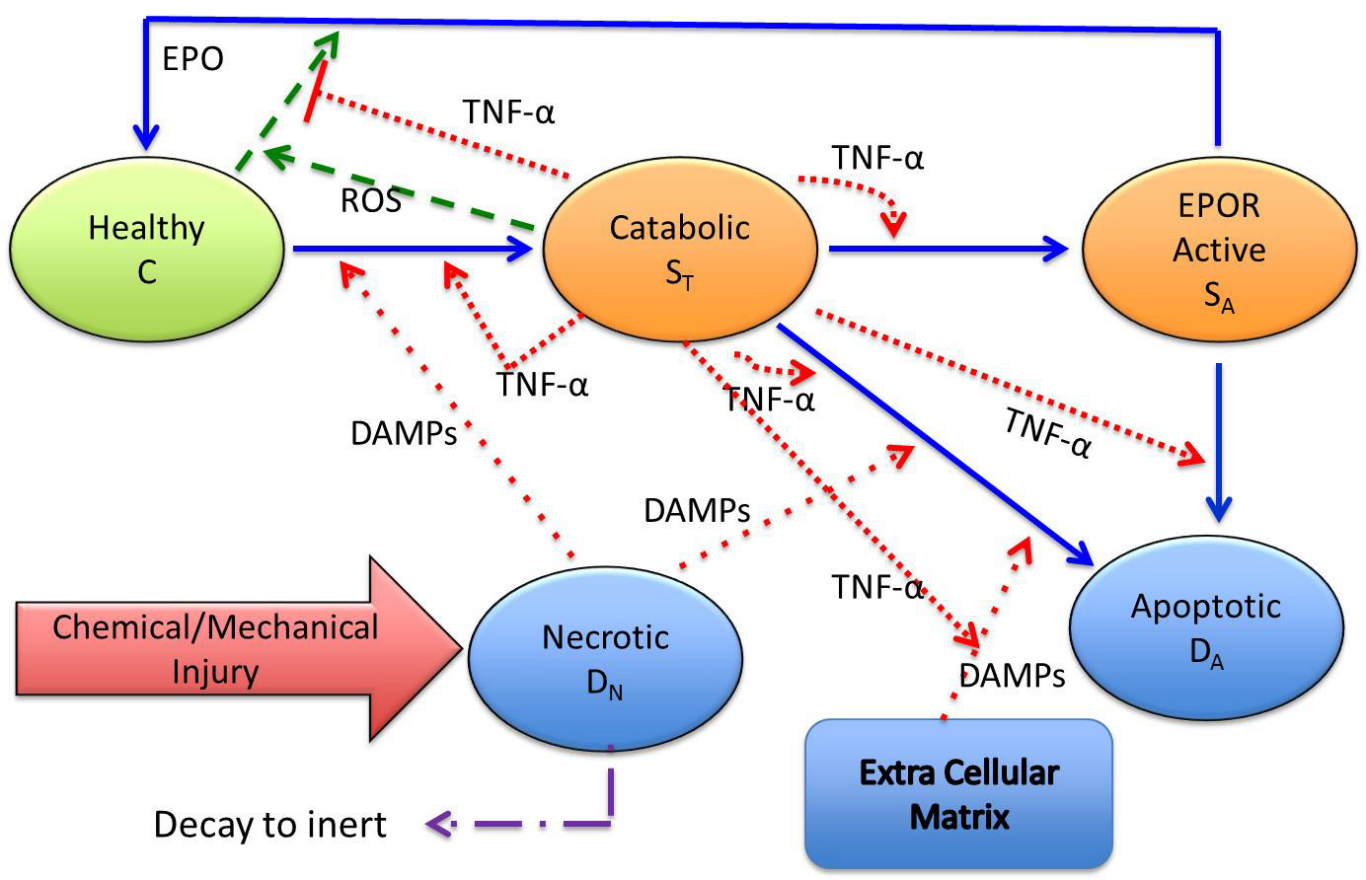
**Signaling involved in cartilage injury response**. The occurrence of an injury begins a sequence of chemical productions that promote the inflammatory response. Lysing (necrotic) cells give off DAMPs (*M*) resulting in a population of catabolic cells that produce TNF-*α *(*F*) and ROS (*R*). ROS promotes production of EPO (*P*) by healthy cells while TNF-*α *acts to block EPO signaling. Furthermore, TNF-*α *influences degradation of extracellular matrix providing another source of DAMPs. The cells switch state according to the signaling by chemicals produced in response to injury and inflammation. DAMPs and TNF-*α *drive the *C *→ *S*_*T *_transition, TNF-*α *drives the *S*_*T *_→ *S*_*A *_transition, while TNF-*α *and DAMPs drive the *S*_*T *_→ *D*_*A *_transition. Finally, EPO drives the *S*_*A *_→ *C *transition. This figure illustrates the assumptions of the mathematical model (equations (3-11)). Not shown: DAMPs given off as a result of matrix degradation also influences the switch from healthy to catabolic.

Figure 4 details the chemical signaling and the switching of chondrocyte cell states represented in the mathematical model presented in the following. An initial injury creates a population of necrotic cells which release alarmins (such as DAMPs) [6,7]. We denote by *M *the concentration of DAMPs at a given time and location. The DAMPs signal healthy cells near the injury to enter the catabolic state resulting in the production of TNF-*α*. We denote the concentration of TNF-*α *at a given time and location by *F*. The inflammatory cytokine TNF-*α *has several effects on the system: It

1. feeds back to continue to switch healthy cells into the catabolic state,

2. causes catabolic cells to enter the EPOR active state [5],

3. influences apoptosis of catabolic and EPOR active cells,

4. degrades extracellular matrix (denoted by *U*) which results in increased concentrations of DAMPs,

5. has a limiting effect on production of EPO [5].

Catabolic cells also produce reactive oxygen species (ROS) which influences the production of EPO by healthy cells. We denote the concentration of ROS at a given time and location by *R*. There is a time delay of 20-24 hours before a healthy cell signaled by ROS will begin to produce EPO.

In developing a mathematical model to represent the scenario described above we assume that the chemicals diffuse throughout a domain. This diffusion is essential in determining the spatial behavior, i.e. the expansion or abatement of the lesion. Since the chondrocytes are fixed in the matrix we do not consider cell motility. First the mathematical model contains four equations (one for each chemical species) describing the dynamics of the chemical concentrations. These equations are each of the form:

(1) $$\begin{array}{c} \text{change in concentration of particular chemical}\\ \;\text{ = diffusion of that chemical}\\ \;\text{ + production of that chemical by cells in appropriate states}\\ \;-\text{natural decay of that chemical}\text{.} \end{array}$$

Next the model consists of four equations for the population densities of cells in the healthy, catabolic, EPOR active, and necrotic states. Each of these equations are of the form:

(2) $$\begin{array}{c} \text{change in population density of particular cell state}\\ \;\text{ = population density of cells switching into that state due to appropriate signaling}\\ \;\text{ - population density of cells switching out of that state due to appropriate signaling}\text{.} \end{array}$$

Finally there is an equation corresponding to the degradation of extracellular matrix by TNF-*α *which feeds back into the production of DAMPs.

The model equations for the concentrations of the chemical species are:

(3) $$\partial_{t}R=\nabla \cdot \left(D_{R}\nabla R\right)-\delta_{R}R+\sigma_{R}S_{T},$$

(4) $$\partial_{t}M=\nabla \cdot \left(D_{M}\nabla M\right)-\delta_{M}M+\sigma_{M}D_{N}+\delta_{U}U\frac{F}{\lambda_{F}+F},$$

(5) $$\partial_{t}F=\nabla \cdot \left(D_{F}\nabla F\right)-\delta_{F}F+\sigma_{F}S_{T},$$

(6) $$\partial_{t}P=\nabla \cdot \left(D_{P}\nabla P\right)-\delta_{P}P+\sigma_{P}C\frac{R\left(t-\tau_{2}\right)}{\lambda_{R}+R\left(t-\tau_{2}\right)}\frac{\Lambda }{\Lambda +F}.$$

The equation describing matrix degradation by TNF-*α *is:

(7) $$\partial_{t}U=-\delta_{U}U\frac{F}{\lambda_{F}+F}.$$

We note that the right hand side of this equation appears in (4) as part of the production of DAMPs as we have assumed that degraded matrix releases alarmins.

The equations for the switching of cell states are:

(8) $$\partial_{t}C=\alpha S_{A}\frac{P}{\lambda_{P}+P}-\beta_{1}C\frac{M}{\lambda_{M}+M}H\left(P-P_{c}\right)-\beta_{2}C\frac{F}{\lambda_{F}+F}H\left(P-P_{c}\right),$$

(9) $$\begin{array}{lll} \partial_{t}S_{T} & =\beta_{1}C\frac{M}{\lambda_{M}+M}H\left(P-P_{c}\right)+\beta_{2}C\frac{F}{\lambda_{F}+F}H\left(P-P_{c}\right)-\gamma S_{T}\left(t-\tau_{1}\right)\frac{F\left(t-\tau_{1}\right)}{\lambda_{F}+F\left(t-\tau_{1}\right)}\; & \text{(1) }\\ & \;-\nu S_{T}\frac{F}{\lambda_{F}+F}\frac{M}{\lambda_{M}+M},\; & \text{(2) }\\ & \; & \text{(3) } \end{array}$$

(10) $$\partial_{t}S_{A}=\gamma S_{T}\left(t-\tau_{1}\right)\frac{F\left(t-\tau_{1}\right)}{\lambda_{F}+F\left(t-\tau_{1}\right)}-\alpha S_{A}\frac{P}{\lambda_{P}+P}-\mu_{S_{A}}S_{A}\frac{F}{\lambda_{F}+F},$$

(11) $$\partial_{t}D_{N}=-\mu_{D_{N}}D_{N}.$$

In (8) and (9) the function *H*(·) is given by

(12) $$H\left(s\right)=\left\{\begin{array}{cc} 1 & \text{if }s<0,\\ 0 & \text{if }s\geq 0. \end{array}\right)$$

The constant *P*_*c *_represents a critical level of EPO above which the effects of DAMPs and TNF-*α *on healthy cells is limited.

Based on the diagram in figure 4 and the assumptions underlying that diagram, one could assume that the function *H*(·) is identically one, i.e. *H *≡ 1. However, if we take *H *≡ 1 then figure 4 implies, and computational results confirm, that there is infinite feedback into the system by alarmins, TNF-*α*, and catabolic cells. As noted in [5] "the pro-inflammatory arm of the injury response is inherently self-amplifying". This does not allow for lesion abatement or limitation of secondary pro-inflammatory cytokine induced injury described in [5]. This suggests that signaling by anti-inflammatory cytokines such as EPO not only promote the switch from the EPOR active state to the healthy state but also influences the response of healthy cells to alarmins and pro-inflammatory cytokines. Thus the mathematical model suggests that in some way the anti-inflammatory cytokines limit the switch from the healthy state to the catabolic state so that there is not an infinite feedback into the system by alarmins, TNF-*α*, and catabolic cells. This is consistent with the observation of Brines and Cerami on the antagonistic relationship between TNF-*α *and EPO, that "each is capable of suppressing the biological activity of the other" [5]. In the model equations (3-11) we take the function *H*(·) as in (12) for convenience. However, there may be a more appropriate form for the function *H*(·) that must be determined through experiment to discover the nature of the effects that sufficient concentrations of EPO or other anti-inflammatory cytokines have on healthy chondrocytes.

We note some features of the mathematical model (equations (3-11)):

1. we have incorporated the time delays for activation of the EPO receptor and synthesis of EPO (6),(9),(10),

2. it requires a concentration of TNF-*α *and DAMPs together for apoptosis of catabolic cells (see (9)),

3. after some time necrotic cells decay to an inert state (see (11)) so that there is not a continuous production of DAMPs for all time from the initial injury. This also corresponds to the loss of cartilage such as is sometimes associated with osteoarthritis.

The specific functional forms appearing in the model system (equations (3-11)) have been chosen to capture the critical thresholds and represent the function shapes qualitatively.

## Results and discussion

Using the mathematical model (equations (3-11)) we can simulate the two scenarios depicted in figure 2. That is, we can manipulate parameter values that correspond first to the case where inflammation is not limited by the presence of anti-inflammatory cytokines (d) and (e) in figure 2 and second to the case where anti-inflammatory cytokines limit the development of secondary inflammation induced injury (d∗) and (e∗) in figure 2. For the computations and simulations we assume a two dimensional dimensional domain. Moreover, as depicted in figure 2 we assume circular symmetry so that changes only occur in the radial direction. In order to simulate the injury response we obtain numerical approximations to the system of partial differential equations (equations (3-11)). This is done by first employing the scheme for discretizing the symmetric diffusion operator from appendix C of [9]. This gives a semi-discrete system of delay-differential equations which are then solved in MATLAB using the dde23 code for solving delay-differential equations. For details on the methods and software for solving delay-differential equations see [10-12]. One of the goals of this model is to provide a conceptual framework for a truly mechanistic understanding of certain aspects of cartilage injury closely related to lesion expansion. This requires biologists to determine parameters experimentally, but we need to know the nature of those parameters before we can go out and measure them. Since many of the parameter values have yet to be determined experimentally, we choose those values to be such that the simulations give quantitatively reasonable cell populations and chemical concentrations. We also choose the parameters so that the expansion or abatement of the lesion occurs over a time period consistent with the experimental results described in [5]. We note that diffusion of the chemical species is the mechanism which drives the expansion of the lesion in our model. The diffusion coefficients have been determined through the methods presented in [13].

**Figure 5:**
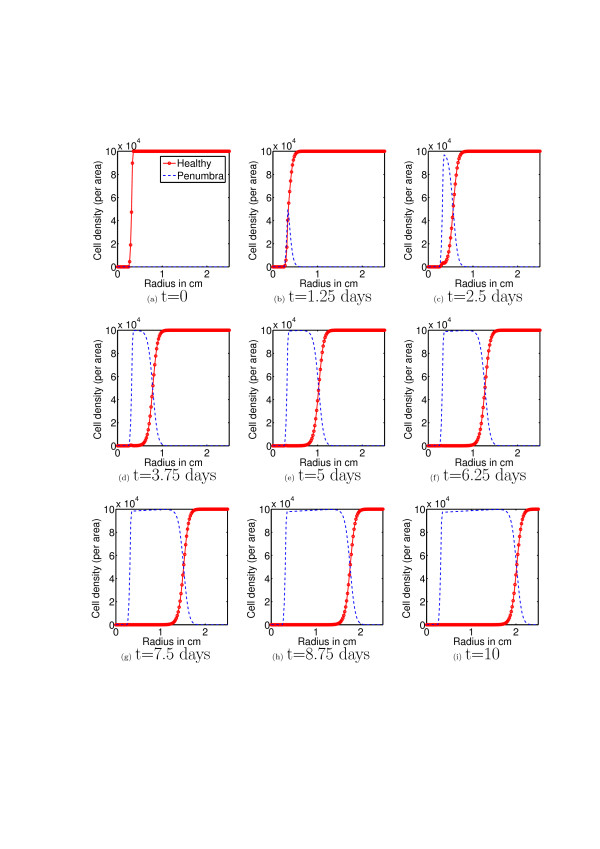
**Healthy and Penumbra cells without EPO**. This figure shows the evolution of the healthy and penumbra (comprised of both catabolic and EPOR active) cell population densities over a period of 10 days. The results shown here correspond to a situation in which there is no production of or response to EPO. We see here that the healthy cell population is decimated. In accord with figure 4 and the corresponding mathematical model (equations (3-11)) this is due to the uninhibited influence of pro-inflammatory cytokines.

**Figure 6:**
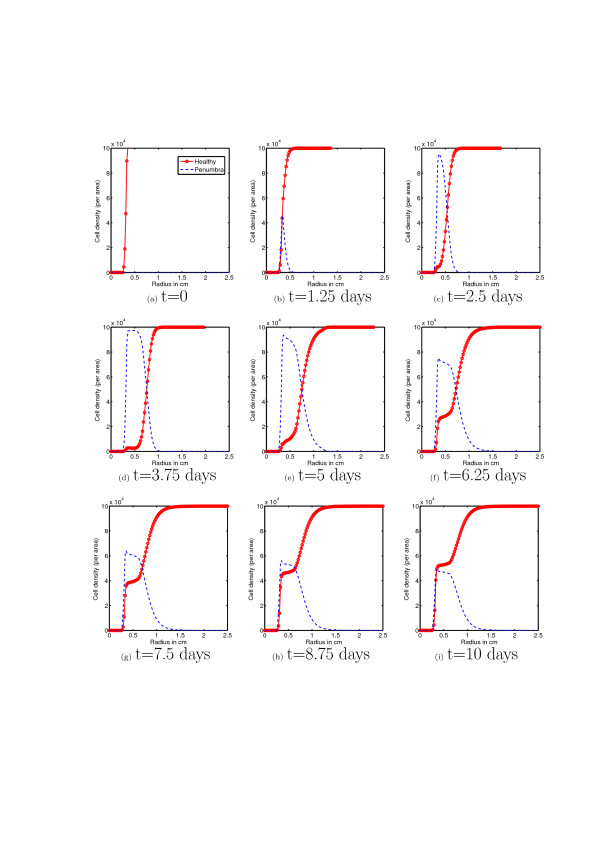
**Healthy and Penumbra cells with EPO**. This figure shows the evolution of the healthy and penumbra cell population densities over a period of 10 days. Initially there is the development of a population of penumbra cells (c) due to the time lags for EPO production and EPOR activation. However, once healthy cells at the edge of the penumbra begin to produce EPO and penumbra cells become EPOR active there is an increase in the population density of healthy cells near the injury and there ceases to be expansion of the penumbra ((d) - (i)).

First, using the mathematical model (equations (3-11)) we simulate the evolution of chondrocyte population density (per area, and assuming circular symmetry, as a function of radius) over a period of ten days after an initial cartilage injury. Figure 5 corresponds to the cascade of events illustrated in the left hand arrows of figure 2, while figure 6 corresponds to the right hand arrows of figure 2. The initial conditions correspond to the presence of an initial injury, i.e. a population of necrotic cells occupying a circle of radius 0.25 mm.

Following the initial injury there is a quick and steady increase in the population density of catabolic and EPOR active cells and decline in the population density of healthy cells (figures 5(b)-(i)). After ten days there has formed a thick penumbra of catabolic cells which are beginning to die off (via apoptosis, see figure 5(i)) showing that without the presence of EPO there is unlimited secondary pro-inflammatory cytokine-induced injury and unabated spread of the cartilage lesion. This corresponds qualitatively well with the expected results illustrated in figure 2(d), (e).

Figure 6 shows the time evolution of chondrocyte population density (per area, and assuming circular symmetry, as a function of radius) over a period of ten days after an initial cartilage injury with the antagonistic effects of EPO on TNF-*α*. The initial conditions are the same as previously described for the simulations without EPO. The dynamics of the chondrocyte cell states population densities are the same over the first day or so as in the case with no EPO. This is due to the time delays for cells to become EPO producing and EPOR active. However, we see by day 3 or so (see figure 6(d) and compare with 6(d)) there is a significant difference in the population dynamics compared with that in the no EPO case. We begin to see a reversion of cells in the EPOR active state back to the healthy state. While there is still some amount of secondary injury due to the inflammatory process and presence of pro-inflammatory cytokines, there is a fixed radius (see figures 6(f)-(i)) beyond which the penumbra, and hence the lesion and secondary proinflammatory cytokine induced injury, cannot expand. Thus there is lesion abatement by the action of the anit-inflammatory cytokine EPO. This corresponds qualitatively well with the expected results illustrated in figure 2(d*), (e*).

Now we describe the behavior of the model under small changes to the parameter values. In order to do so we vary the parameters one at a time for a small range and observe how this effects the behavior of the healthy and penumbral cells. This exploration of the parameter space gives some indication of which parameters dominate the dynamical behavior for the model equations. For a given parameter *z *we vary the value over a range of [0, 2*z*], with the exception of *λ*_*R*_, *λ*_*M*_, *λ*_*F*_, *λ*_*P*_, *α *which must be strictly positive. Here we focus on those parameters associated with nonlinear terms in the model equations. Table 3 summarizes the results described in what follows. Note that below and in table 3 when we say "robust" we mean that the qualitative behavior of the simulations in unchanged as the parameter value is varied. We first consider varying the values of *σ*_*R*_, *σ*_*M*_, *σ*_*F*_, *σ*_*P*_, i.e. the production rates for each of the four chemical species. For both the production of ROS, *σ*_*R*_, and the production of EPO, *σ*_*P*_, decreasing these values leads to simulations which steadily approach the case with no EPO. That is, lesion expansion becomes more and more pronounced until inflammation and secondary cytokine induced injury become unchecked. This behavior is as to be expected. In contrast, increasing the values for these two parameters results in the abatement of lesion expansion at earlier times. Increasing or decreasing the production rate, *σ*_*F *_of TNF-*α *has the exact opposite behavior to increasing or decreasing *σ*_*R *_or *σ*_*P*_. For positive values of the production rate of DAMPs, *σ*_*M*_, the long term behavior of the simulations are stable. This is because the principal role of alarmins is to initiate inflammation and thus are most influential for early time. Setting *σ*_*M *_= 0 results in no inflammation which is as to be expected. Next we note two interesting features of the simulations which occur when the values of *α*, and *γ *are decreased. As the values of these two parameters are decreased, we observe the formation of a penumbra of smaller and smaller radius. However, the cells forming the penumbra are much slower to respond to EPO. This makes sense for the parameter *α *since this is the rate at which EPOR active cells convert back to the healthy state. On the other hand, γ is the rate at which catabolic cells switch to the EPOR active state. In this case, small values of *γ *correspond to a situation in which the penumbra is made up of predominantly catabolic cells which do not respond to EPO. We note that increasing *λ*_*R*_, *λ*_*F *_has the same effect as decreasing *σ*_*R*_, *σ*_*F *_and similar for decreasing *λ*_*R*_, *λ*_*F*_. Also varying *λ*_*M *_has an expected effect since it is the saturation constant for DAMPs. Finally, the other parameters are robust as noted in table 3.

**Table 3:** Response of cells to small perturbations in parameter values

Response of cell behavior to small changes in parameter values		
**Parameter**	**Response to increase in value**	**Response to decrease in value**

*σ*_*R*_	Lesion expansion is abated sooner	Steadily approaches no EPO case

*σ*_*M*_	Robust	Robust, until *a*_*M *_*= *0 when no inflammation occurs

*σ*_*F*_	Lesion abatement becomes less efficient	Lesion expansion is abated sooner

*σ*_*P*_	Lesion expansion is abated sooner	Steadily approaches no EPO case

Λ	Robust	Robust (must have Λ > 0)

*λ*_*R*_	Lesion abatement becomes less efficient	Lesion expansion is abated sooner

*λ*_*M*_	Lesion expansion is abated sooner	Steadily approaches unabated lesion expansion

*λ*_*F*_	Lesion expansion is abated sooner	Lesion abatement becomes less efficient

*λ*_*P*_	Robust	Robust

*α*	Robust	Penumbra becomes more vigorous but smaller in radius

*β*_1_	Lesion abatement becomes less efficient	Formation of penumbra steadily decreases

*β*_2_	Robust	Robust

*γ*	Robust	Penumbra becomes more vigorous but smaller in radius

*ν*	Robust	Robust

$\mu_{S_{A}}$	Robust	Robust

$\mu_{D_{N}}$	Robust	Robust

This tables summarizes the results of the parameter study described in the main body of the text. When we say "robust" we mean that the qualitative behavior of the simulations in unchanged as the parameter value is varied.

## Conclusions

Based on the biological assumptions summarized in the diagram shown in figure 2 we have developed a mathematical representation of some of the principal features of the injury response in articular cartilage. In particular we have captured, qualitatively, secondary pro-inflammatory cytokine induced injury and lesion based on the balance between TNF-*α *and EPO as described in Brines and Cermani [5]. This modeling effort suggests that anti-inflammatory cytokines may act in such a way as to influence the response of chondrocytes to alarmins and pro-inflammatory cytokines. In particular sufficient concentrations of antiinflammatory cytokines may limit healthy chondrocytes conversion to the catabolic state. Provided dosages, dosage responses, delivery method, etc. the model (equations (3-11)) can be applied to study the effectiveness of the introduction of exogenous EPO as a potential therapy for limiting secondary pro-inflammatory cytokine induced injury and promoting healing in articular cartilage. We note however, that due to the time delays for cells to become EPOR active, there is little or no response to EPO at early times after an injury has occurred. Thus there is a window of time during which introduction of tissue-protective EPO derivatives such as discussed in [5] are ineffective as therapies. This is evidenced by comparing figures 5(a)-(b) with figures 6(a)-(b) in which there is little or no difference between the case with EPO and the case without. The model presented herein also has potential applications for studying other issues related to post-traumatic stress in articular cartilage. By coupling this model with mechanical models, or including geometric features of cartilage one may be able develop a more complete theoretical description of aspects of cartilage injury of relevance in the biomedical sciences.

## Competing interests

The authors declare that they have no competing interests.

## Authors' contributions

All authors read and approved the final manuscript. JMG carried out the simulations and drafted the manuscript. JMG BPA PSR JAM developed the mathematical model and interpreted the results of the simulations. LD performed cell blot experiments.

## Reviewer Comments and Responses

Reviewer number 1: Yang Kuang

The authors claim to present a minimal mathematical model based on known mechanisms to investigate the spread and abatement of such lesions. This is debatable as the model involves both diffusion and time delay yet the simulation does not address the spatial structure. The simulated dynamics is rather simple which suggests that some simple nonlinear system of ODEs may generate comparable outcomes. While modeling anything biological of this complexity will inevitably involve many simplifying assumptions and even the plausible models are ad hoc, I think the authors can present much more details on their model formulation and validation. Why can not some simple ODE models accomplish what they did here? The authors mentioned that specific functional forms appearing in their model system (equations (3-11)) have been chosen to capture the critical thresholds and represent the function shapes qualitatively. Later the authors refer that information as figure 2 which is hardly illustrative for thresholds or function shape. It is also important to provide meaningful parameter estimation and the source of information or the lack of it.

Response to review 1:

Reviewer 1 mentions that the simulated dynamics do not address the spatial structure, but the simulations (Figures 5 and 6) show time slices of radial profiles cell populations which corresponds to the radial expansion of a lesion. Thus the authors believe that the simulations do address spatial structure. Moreover, diffusion of cytokines is the principal mechanism driving the expansion of lesions in articular cartilage injuries so that omitting diffusion ignores a principal mechanism behind one of the facets of cartilage injury response that is of primary interest. We would also like to clarify the inclusion of time delays in our model. Delays are included since, as described in the biological literature, there are time lags between when cells are signaled to become either EPOR active and/or EPO producing. By using delay terms rather than ODE's that capture the lesion abatement process phenomenologically, we maintain a clear connection between model parameters and the underlying physiology.

Reviewer 1 brings up a concern regarding the parametrization of the model. Reviewer 2 also has similar concerns so we address both simultaneously. First, in the revised manuscript we have provided a table listing the parameter values for the mathematical model used in the simulations. A small number of these parameters have been determined from literature which is cited in the revised manuscript. The remaining parameters have been approximated to give reasonable results. By this we mean cell population densities and chemical concentration levels that are consistent with those observed in other, but not necessarily injury related, cartilage experiments. Also we choose approximated parameter values that give lesion expansion and abatement occurring over a time frame as is observed experimentally. Second, one of the goals of our model is to provide a conceptual framework for the orthopaedics community to understand this phenomena from the perspective of cytokine interactions and determine what information is missing, so that we can eventually have a truly mechanistic understanding of lesion abatement. Moreover, we have attempted to develop a model that will motivate additional experiments to determine parameters, since to our knowledge many of the parameter values for this type of mechanistic model have yet to be carried out. We feel that it is of value to have these parameter values determined experimentally rather than employing statistical parameter estimation techniques. The level of additional parametrization called for in this modeling effort is consistent with other similar models in the literature, for an example see the recent article [14].

Reviewer number 2: James Faeder

### Summary

This paper presents a model of inflammation and tissue damage in cartilage. The model considers secretion and diffusion of soluble inflammatory and anti-inflammatory mediators and their effects on tissue, which is modeled as a set of spatial continuum variables. Time courses of tissue populations (healthy vs. dead or damaged cells) are determined for two different scenarios starting from the same initial damage - first with negative feedback from anti-inflammatory mediators and second with anti-inflammatory mediators. The simulations show that negative feedback from anti-inflammatory mediators is necessary for recovery of damaged tissue to occur. Some experimental results from cell culture experiments are presented in the Background section and show that some effects of inflammation can be mitigated by EPO, which provides support for a key assumption of the model. Major comments 1) Missing parameters. Although the structure of the model is reasonably well described, unless I somehow missed them model parameters are not given in the paper. This should be required for publication. Parameters need to be provided along with some discussion of how they were chosen and references supporting those choices. 2) The results seem very preliminary. No attempt is made to compare the results of the simulation with experiments. Results are only presented for two scenarios and no characterization of the effect of varying model parameters is given. Have wound recovery experiments been performed, for example, in which the anti-inflammatory response is somehow manipulated - either enhanced or suppressed? Can the behavior of the model be modulated by changing various parameters that could either vary or be manipulated in actual wounds? The main point seems to be that without anti-inflammatory responses acting as a negative feedback, the inflammatory response, which has built-in positive feedbacks, will proceed unchecked. Although it is useful to a degree to demonstrate that the expected behavior does occur for a realistic model (something that has not completely been shown here because of point (1)), I think that is just the jumping off point for a reasonable model and does not by itself justify publication. As the paper stands currently, I do not think there is enough here to warrant a journal publication. Minor comments p. 2. The background starts immediately with presentation of results in Figure 1 without sufficient motivation. p. 4. "We assume chondrocytes become necrotic only at the time of an initial injury." What is the basis for this assumption? p. 5 and Figure 4. The diagram is somewhat confusing. TNF-alpha is shown as stimulating ECM, whereas in the model it stimulates degradation of ECM. Why are the DAMPs released by ECM degradation shown only as increasing apoptosis of ST cells and not also of healthy cells (or EPOR active cells). p. 6. Eq. 6. Is the mathematical form of the delay equation correct for the physical scenario? The production of EPO at the current time is assumed to be proportional to the population of healthy cells at a time *τ*_2 _earlier (taken to be 24 hours), but what if the healthy cell population is rapidly decaying during that time? It would seem that the healthy cells that become catabolic cells should not be producing EPO. p. 8. Solution of partial differential equations for system with circular symmetry. References given are for numerical solution of ordinary diff. eqs., but the scheme for solving the PDEs is not described. A one or two sentence description and a reference should be given for the scheme used.

Response to review 2:

As reviewer 2 points out, the experimental results discussed in this manuscript are presented in the background without motivation. In the revised manuscript we have tried to briefly motivate these results.

Reviewer 2 has asked for a justification for our assumption that chondrocytes become necrotic only at the time of initial injury. Our statement in the manuscript quoted by reviewer 2 is a little ambiguous. What we meant is that the initial injury results in cell death through necrosis, while secondary cytokine-induced injury results in cell death through apoptosis. While these may be simplifying assumptions, it is not entirely clear which type of cell death dominates in osteoarthritis. Moreover, we have provided an additional reference to lend some support to our assumptions and have clarified the quoted statement in the manuscript with a more detailed discussion.

Reviewer 2 has pointed out that Figure 4 is somewhat confusing, in that it is difficult to tell from the diagram that TNF-*α *degrades the extracellular matrix (ECM). Although, it is clearly stated in the caption to that figure that this is the case, and the mathematical model reflects the figure is confusing. We have modified the diagram shown as Figure 4 in the manuscript to try to make it more clear that extracellular matrix is not stimulated by TNF-*α *but is a "catalyst" of sorts of DAMPs due to degradation of ECM by TNF-*α*. DAMPs influence cells in different states in different ways. DAMPs do not cause healthy cells to enter directly into apoptosis. Healthy cells signaled by alarmins must first become "sick", i.e. catabolic, before they can enter into apoptosis.

Reviewer 2 has pointed out an error in the form of the production term for EPO. The production of EPO should be proportional to the healthy cell population at the current time, not at a *τ*_2 _earlier time. We have modified the equations in the revised manuscript and carried out and included new simulations. The results are qualitatively the same, however in the second scenario it takes a slightly longer time for lesion abatement to occur since there are fewer (at least near the wound) healthy cells to produce EPO. We note that in the first scenario we take EPO production to be zero so that there is no different from the results in the previous version of the manuscript.

Reviewer 2 has pointed out that we neglected to describe the scheme for discretizing the partial differential equations that make up our mathematical model in carrying out numerical simulations. We have addressed this by stating the scheme used and providing a reference for this scheme.

Reviewer 3: Anna Marciniak-Czochra

Provided no comments for publication.
